# Specific Changes in the Mammalian Gut Microbiome as a Biomarker for Oxytocin-Induced Behavioral Changes

**DOI:** 10.3390/microorganisms9091938

**Published:** 2021-09-12

**Authors:** Itzhak Dangoor, Dušanka Stanić, Leah Reshef, Vesna Pešić, Uri Gophna

**Affiliations:** 1 The Shmunis School of Biomedicine and Cancer Research, The George S. Wise Faculty of Life Sciences, Tel Aviv University, Tel Aviv 6997801, Israel; itzik.dangoor@gmail.com (I.D.); leahfa@gmail.com (L.R.); 2 Abarbanel Mental Health Center, Bat Yam 5943602, Israel; 3 Center for Experimental and Applied Physiology, Department of Physiology, Faculty of Pharmacy, University of Belgrade, 11000 Belgrade, Serbia; dusanka.stanic@pharmacy.bg.ac.rs (D.S.); vepesic@gmail.com (V.P.)

**Keywords:** microbiome, oxytocin, biomarker, psychiatry, gut-brain axis

## Abstract

Prolonged exposure to psychiatric pharmacological agents is often associated with marked gastrointestinal phenomena, including changes in food intake, bowel motility, gastric emptying, and transit time. Those changes are reflected in the gut microbiota composition of the patient and can, therefore, be objectively measured. This is in contrast to the standard psychiatric evaluation of patients, which includes symptoms that are subjectively assessed (i.e., mood, anxiety level, perception, thought disorders, etc.). The association between a drug’s effect on the microbiota and psychiatric symptoms may allow for quantifiable surrogate markers of treatment effectiveness. Changes in the levels of specific drug-sensitive bacterial species can, thus, potentially serve as biomarkers for the intake and effectiveness of psychiatric drugs. Here, we show substantial microbiota changes that were associated with oxytocin administration and the decreased anxiety/depression-like behaviors it conferred in a rat model of corticosterone-induced stress. Compared with oxytocin, citalopram produced more minor effects on the rats’ microbiota. Alterations in the gut microbiota may, therefore, reflect the consumption and effectiveness of some psychiatric drugs.

## 1. Introduction

Humans are colonized by bacteria, fungi, archaea, and viruses, which are collectively referred to as the microbiome. Most of the microbiome resides in the gut and may easily be investigated via stool sampling and subsequent metagenomic DNA sequencing. Prolonged exposure to psychiatric pharmacological agents is often associated with marked gastrointestinal phenomena, including changes in food intake, bowel motility, gastric emptying, and transit time [1,2,3]. Unlike the relatively objective measurement of the microbiota composition, accurate assessment of patients’ therapy adherence and treatment outcomes represent a challenge in psychiatric medical care [4]. This is partly because, for most psychopharmacological agents, compliance and response to treatments are subjectively assessed based on self-reporting and physicians’ evaluations [5,6]. An interesting alternative is having changes in the psychiatric patients’ gut microbiota composition serve as a measurable proxy for monitoring patients’ compliance and the therapeutic effects of some drugs.

It is yet unclear how behavioral changes and drug intake affect the microbiota; however, mounting evidence suggests that physical and mental disturbances may lead to changes in gastrointestinal (GI) motility [7,8] in both animals and humans [9,10,11]. Indeed, in humans, anger, fear, pain, and anxiety, as well as intensive exercise, results in changes in GI activity [8]. In rats, chronic stress results in initial delayed gastric emptying followed by acceleration later on [12]. Medication intake [13,14] and changes in stool consistency, gastric transit, and emptying time [15,16] also have a great impact on microbial composition. Previous microbiota studies have already shown a connection between the microbiota and behavior, with the association of relative abundance of specific bacteria to stress exposure and anxiety [17,18,19,20,21]. However, the association of microbial changes with additional factors such as diet [22,23] and physical activity [24] limits the current use of the microbiota as a biomarker. Nevertheless, an increasing amount of evidence suggests that the ‘gut brain axis’ has a significant role to play in stress response [25,26,27]. This is especially evident by the high sensitivity of the microbiota to the effect of stress [28,29,30] and by studies demonstrating a correlative link of the microbiota composition with stress in both rodents [31,32,33,34] and humans [35,36,37].

The degree of change in the microbiota composition between time points is sometimes defined as volatility [38,39]. A recent study has concluded that gut microbial volatility is influenced by stress since mice with a greater stress response exhibited a greater microbial volatility. Furthermore, microbial volatility was positively correlated with cortisol and corticosterone after chronic stress in humans and mice, respectively [40]. Another study conducted on patients diagnosed with depression and anxiety showed that volatility was different among patients and was affected by the type of drug used in their treatment [41]. Based on these recent studies, it is reasonable to propose that a large microbial volatility or changes in specific taxa could sometimes indicate that certain psychiatric drugs have had a physiological effect. To test whether microbiota can serve as a biomarker for treatment success, we investigated the degree of microbiota composition change in response to drug administration, as well as the correlation to behavioral parameters. We defined the degree of fecal microbiota change as *microbiota composition shift* and tested it as a possible biomarker predicting drug intake and success. Three agents known to elicit behavioral changes in rats were investigated for their effects on the rats’ stool microbiota. These included corticosterone, an anxiogenic agent [42]; citalopram, a well-tolerated antidepressant and anxiolytic agent of the SSRI (selective serotonin reuptake inhibitor) family [43,44,45], previously shown to influence gut motility [46]; and oxytocin, a naturally produced neuropeptide and a potential anxiolytic-like agent [47].

## 2. Materials and Methods

All animal experiments were conducted at the Faculty of Pharmacy of Belgrade and were approved by the Committee for ‘Ethical Animal Care and Use’ of the Faculty of Pharmacy University of Belgrade (Permit Number: 323-07-00067/2015-05), which acts in accordance with the NIH Guide for the Care and Use of Laboratory Animals, 1996.

### 2.1. Animal Housing and Samples Collection

**Figure 1:**
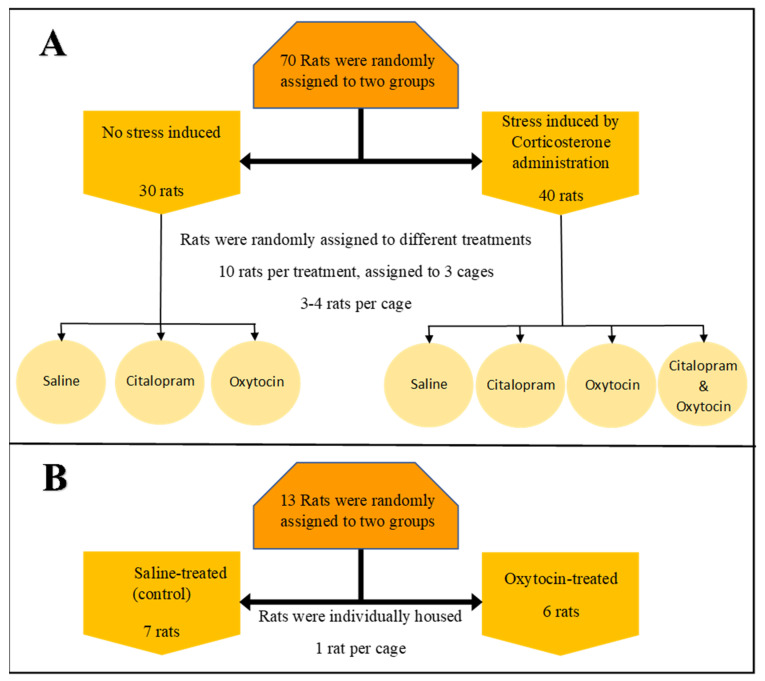
Experimental setup. All rats were 8-week-old male Wistar rats weighing 250–300 g. After a 4-day adaptation period, the rats were randomly divided into experimental groups. Fecal samples were collected randomly from each cage on day 1 and day 20 of the experiment. Saline citalopram or oxytocin was given from day 7 to day 21 by subcutaneous administration and was injected at the same time every day (9:00–10:00 a.m.): (**A**) First experiment, multidrug environment. Stress was induced by non-invasive drinking water-mediated corticosterone administration for 21 days. (**B**) Second experiment, single drug environment.

Eight-week-old male Wistar rats (Military Farm, Belgrade, Serbia) weighing 250–300 g were housed in groups at a constant temperature of 20 ± 2 °C in a 12-h light/dark cycle, with ad libitum access to water and commercial rat food. The bedding was changed three times per week. After a 4-day adaptation period, the rats were randomly divided into the experimental groups described in Figure 1. Fecal samples were collected from each cage on day 1 and day 20 of the experiment. Gloves and sterile tweezers were used for the collection of the samples. After each sampling, 70% ethanol was used for the re-sterilization of tweezers. Upon collection, samples were placed in sterile Eppendorf tubes (2 mL) and stored at −20 °C.

#### 2.1.1. Multidrug Environment: Experimental Setup—Experiment 1

Seventy rats were randomly assigned to experimental groups, as detailed in Figure 1. Stress was induced by chronic administration of corticosterone (Sigma-Aldrich Co., Cat. No. C2505, St. Louis, MO, USA), dissolved in tap water containing 2% polyoxyethylene glycol sorbitan monooleate (Tween 80^®^) to a final concentration of 100 mg/L for 21 days [42]. Non-invasive drinking water-mediated corticosterone administration was used to ensure continual exposure to the elevated hormone level [48]. Citalopram HCL, 10 mg/kg BW, oxytocin at 10 IU per animal, or pure saline were freshly dissolved in 400 uL saline and given on day 7 to 21 by subcutaneous administration injected at the same time every day (9:00–10:00 AM). The control group received drinking water containing 2% Tween 80^®^ for 21 days.

#### 2.1.2. Single Drug Environment: Experimental Setup—Experiment 2

Thirteen rats, individually housed, were randomly assigned into two experimental groups, oxytocin-treated (six rats) and oxytocin-free (seven rats), as detailed in (Figure 1). Drug administration and sample collections were according to the setup of Experiment 1.

### 2.2. DNA Extraction and Microbiota Analysis

Total DNA from the fecal samples were extracted using the ‘PowerSoil DNA isolation kit (MOBIO))’ according to the manufacturer’s protocol. PCR amplification of the bacterial 16 S rRNA gene was carried out with universal prokaryotic primers (containing 5’-end common sequences and their adaptors) (CS1-341F 5’-ACACTGACGACATGGTTCTACANNNNCCTACGGGAGGCAGCAG and CS2-806R 5′-TACGGTAGCAGAGACTTGGTCTGGACTACHVGGGTWTCTAAT). Reactions were prepared in a dedicated PCR cabinet with a filtered air laminar flow, using the Kappa2G DNA polymerase and the following PCR conditions: 3 min in 95 °C, 25 cycles of 15 s in 95 °C, 15 s in 53 °C, and 15 s in 72 °C. Negative controls containing no template were included to verify lack of contamination. Paired-end DNA sequencing was performed on the Illumina MiSeq platform at the Chicago Sequencing Centre of the University of Illinois. Sequencing depth ranged from 12,335 to 26,603 seqs/sample for Experiment 1, and 1237 to 46,999 seqs/sample for Experiment 2. To ensure data evenness, the data were rarefied to the depth of the lowest sample.

Initial merging of paired end reads and quality filtering was performed according to the human microbiome project guidelines (http://www.hmpdacc.org/, accessed on 12 September 2019). Sequences were then analyzed using the Quantitative Insights Into Microbial Ecology (QIIME) package [49]. Usearch 6.1 [50] was used for chimera detection and removal; OTU picking (0.99 similarity) and taxonomy assignment were conducted using the UCLUST algorithm and Greengenes 13.8.2013 database version as a reference. Further identification of bacterial species was performed using the NCBI BLAST [51] algorithm. Abundance weighted and unweighted UniFrac distance matrices, obtained from QIIME, were exported to PAST [52], a statistical analysis program in which principle coordinate analysis (PCoA) and analysis of similarity (ANOSIM) were performed. LDA effect size estimation (LEfSe [53]) was applied to identify which bacterial taxa contribute most to the differences between the two groups. The statistical significance threshold was α = 0.05 for all tests.

### 2.3. Behavioral Data

Behavioral data were collected only in Experiment 1 (see below) using both the elevated plus maze test (EPM), in which anxiety-like behavior is expressed by the animal spending more time in the mazes’ enclosed arms), and the open field test (OF), in which anxiety-like behavior is expressed by a decrease in rat locomotor activity and preference for field edges. While behavioral tests were conducted per individual rat, the microbiota data were collected per cage. Therefore, to compare these two variables, the mean behavioral test score per cage was used.

## 3. Results

### 3.1. Oxytocin Has a Strong Impact on the Composition of Rat Fecal Microbiota

We used a previously established rat model for chemically induced stress [54] to explore the influences of stress induction and anxiolytic modulation on the rats’ gut microbiota in a multidrug environment.

Rats were divided into treatment groups as described in Figure 1. In brief, 12 cages (housing 3–4 rats each) were continuously stress-induced by corticosterone for 21 days. The anxiolytic/anti-depressive drug citalopram, oxytocin, a hormone with anxiolytic-like properties, or both were administered by daily injections between day 7 and 21. To control for the injection effect, three cages received an injection of saline only. An additional nine cages were kept stress-free (no corticosterone was administered) but received anxiolytic/control injections (either oxytocin, citalopram, or saline). Fecal samples were taken for microbiota analysis pre- and post-treatment (see Materials and Methods).

**Figure 2:**
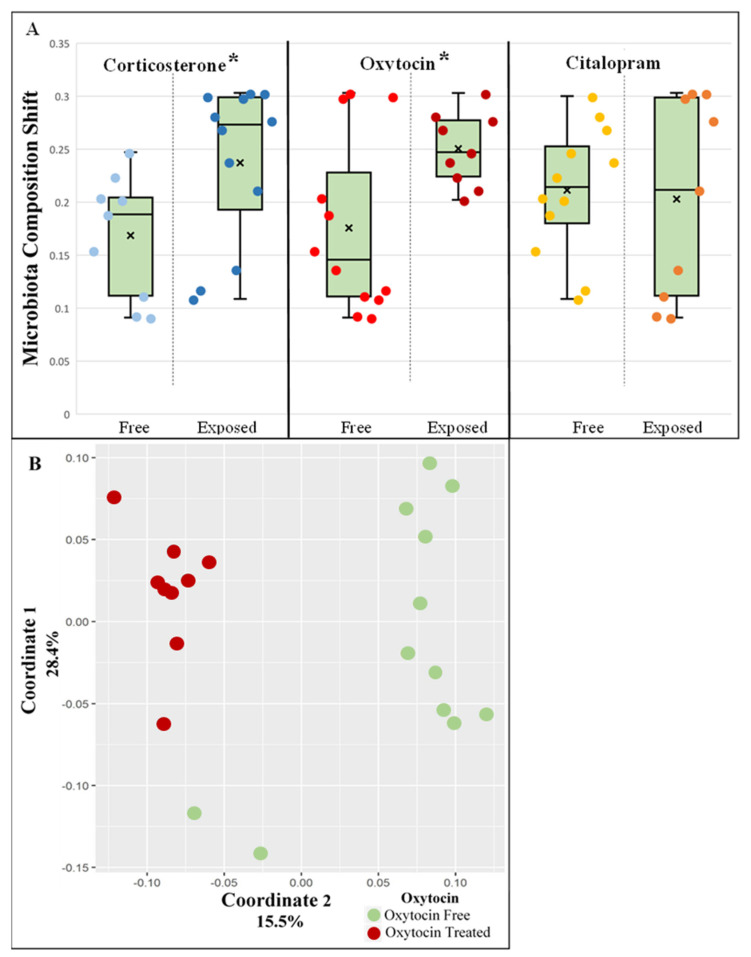
Treatment effect on microbiota: (**A**) the microbiota composition shift (unweighted UniFrac distances between phase I (pre-treatment) and phase II (post-treatment) microbiota samples) *n*; each point represents a single cage. * Significant *p*-value (<0.05); (**B**) PCoA of the unweighted UniFrac matrix of all—post-treatment samples. ANOSIM, *p* = 0.001, R = 0.6.

To better understand the effect each treatment had on the microbiota composition in a multidrug environment, samples were analyzed by 16 S rRNA gene amplicon sequencing and grouped by individual treatment. To overcome the overlap in the treatment groups, the analysis was repeated three times, each employing a different grouping variable (i.e., citalopram, oxytocin, or corticosterone status). Consequently, groups with a minimum of nine samples were obtained, with a single common treatment differentiating them from the rest of the samples. The pre- and post-treatment effect of the degree of clustering was observable when the unweighted UniFrac distance matrix was visualized using PCoA for corticosterone (ANOSIM, *p* = 0.003, R = 0.23) and more so for oxytocin (ANOSIM, *p* = 0.001, R = 0.54), but not for citalopram (Supplementary Figure S1A–C). Pairwise before/after unweighted UniFrac distances between samples were computed to assess which treatment had the largest effect on the microbiota, i.e., led to the largest change in microbial composition between experimental phase I (pre-treatment) and phase II (post-treatment). Both oxytocin treatment and corticosterone stress induction caused significant changes in bacterial composition (Kruskal–Wallis Test; corticosterone, *p* = 0.023; oxytocin, *p* = 0.047). No such change was observed with citalopram (Figure 2A). Similar trends were observed when abundance weighted UniFrac distances were used, but in this case, the results did not reach statistical significance (Supplementary Figure S2). The degree of change in the rats’ fecal microbiota pre- and post-treatment was thereafter defined as the ‘microbiota composition shift.’

The effects of different treatments were also evident when the entire phase II (post-treatment) unweighted UniFrac distance matrix was visualized using PCoA. The separation between oxytocin-treated and untreated animals was strong and statistically significant (Figure 2B; ANOSIM, *p* = 0.001, R = 0.6). Corticosterone induced a lesser yet significant effect on the microbiota (Supplementary Figure S3A; ANOSIM, *p* = 0.001, R = 0.43). No significant effect was observed when cages were grouped according to citalopram (Supplementary Figure S3B). To summarize, oxytocin seems to strongly affect the rat fecal microbiota in both stressed and non-stressed animals.

**Figure 3:**
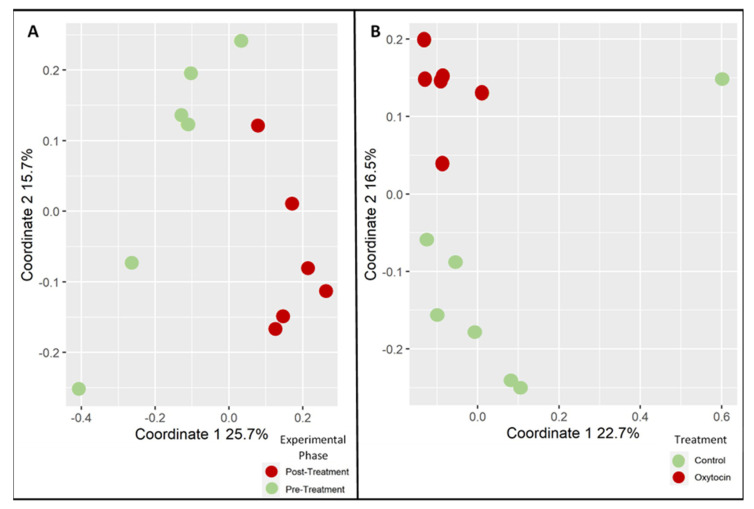
Oxytocin effect in a single-treatment validation experiment. Each point represents the microbiota composition of a single rat: (**A**) samples from the oxytocin-treated group only; pre-treatment (green) vs. post-treatment (red) ANOSIM, *p* = 0.002, R = 0.63; (**B**) all post-treatment samples, oxytocin-treated (red) or saline-treated (green) ANOSIM, *p* = 0.003, R = 0.44.

A second experiment was performed in order to validate oxytocin’s effects as a single drug. Thirteen individually housed rats were divided into treatment groups, as described in (Figure 1). In brief, oxytocin or saline was administered by daily injections between day 7 and Day 21. Fecal samples were taken for microbiota analysis pre- and post-treatment (see Materials and Methods). The ANOSIM results for the unweighted UniFrac analysis were consistent with the first experiment, thereby showing that oxytocin treatment has a strong and significant effect on the rats’ microbiota. This was evident when testing the degree of clustering according to the experimental phase (pre- and post-treatment) in the oxytocin-treated group (Figure 3A; ANOSIM, *p* = 0.002, R = 0.63), as well as when comparing the degree of clustering according to treatment (oxytocin or saline) in all phase II samples (Figure 3B; ANOSIM, *p* = 0.003, R = 0.44).

### 3.2. Changes in Relative Abundance of Specific Bacterial Taxa

**Figure 4:**
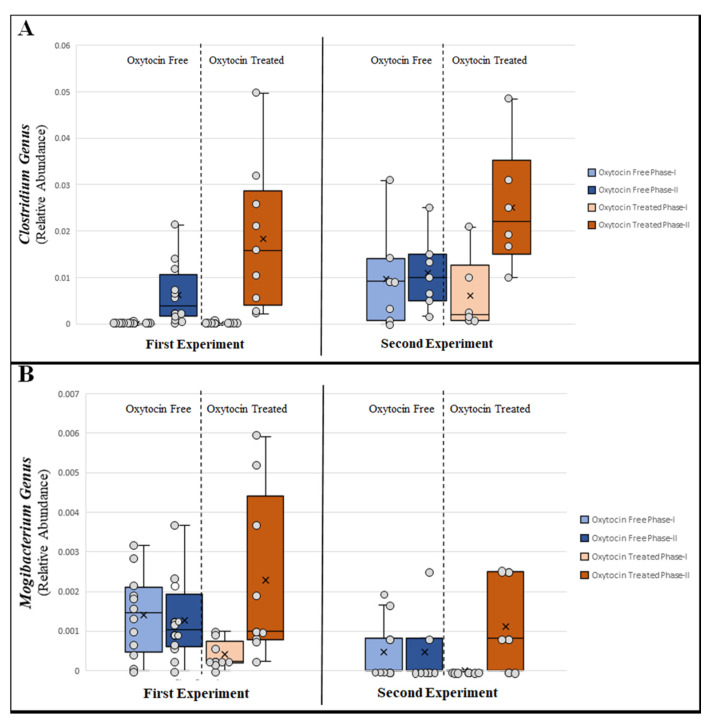
Bacterial genera significantly differentiating oxytocin-treated and non-treated groups: first experiment, each circle represents a single cage pool; second experiment, each circle represents a single animal; phase I, pre-treatment; phase II, post-treatment; (**A**) *Clostridium*; (**B**) *Mogibacterium*.

We next attempted to identify which *specific* bacterial taxa drove the shift in microbial composition between oxytocin-treated and untreated animals. To this end, the widely used, LDA-based LEfSe [53] algorithm was applied. Seventeen taxa in the first experiment and 12 in the second were identified as significantly contributing to the microbial shift between oxytocin-treated and oxytocin-free samples. Four bacterial genera were consistently identified across both independent experiments. Of those, *Clostridium*, *Collinsella*, and *Eubacterium* tended to increase over time in all treatment groups. However, only in the oxytocin-treated group was that increase strong and statistically significant (Figure 4A, Supplementary Figure S4A,B). The genus *Mogibacterium* proved to be the most interesting since it increased solely following oxytocin treatment, while its relative abundance in the other treatment groups decreased over time (Figure 4B).

### 3.3. Specific Bacteria Are Associated with Changes in Behavior

**Figure 5:**
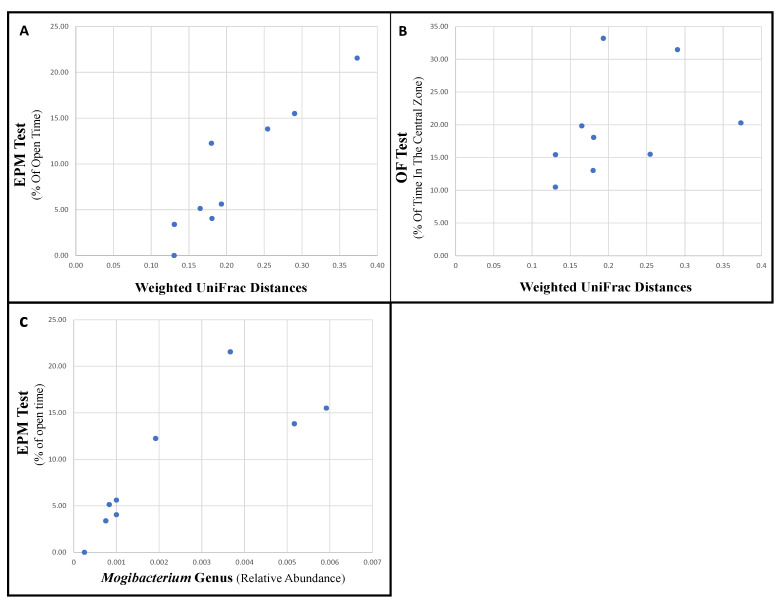
Correlation of behavioral test scores with microbiota parameters in oxytocin-treated rats. The cage mean behavioral tests score was used. Spearman’s correlation values: (**A**) weighted UniFrac Distance and EPM test, *p* = 0.001, R = 0.92; (**B**) weighted UniFrac Distance and OF test, *p* = 0.04, R = 0.7; (**C**) relative abundance of *Mogibacterium* and EPM, *p* = 0.001, R = 0.92.

Since oxytocin alone had a significant, directional, and consistent impact on the microbiota composition, samples from oxytocin-treated rats were chosen for analyzing the possible link between that treatment, microbiota changes, and rat behavior. For comparisons with the microbiota, we used the cage mean behavioral test scores. The microbiota composition shift (pairwise-weighted UniFrac distances pre- and post-treatment) was strongly and significantly associated with the change in behavioral tests scores mediated by oxytocin (Spearman’s ρ for EPM Test parameters: ‘% of Open Time,’ r = 0.92, *p* = 0.001; ‘% of Open Entries,’ r = 0.92, *p* = 0.001; ‘Number of Total Entries,’ r = 0.75, *p* = 0.03; ‘Closed Arm-Entries,’ r = 0.6, *p* = 0.09; Figure 5A) (Spearman’s ρ for OF Test parameters: ‘% of Time in the Central Zone,’ r = 0.7, *p* = 0.04; ‘% of Distance in the Central Zone,’ r = 0.7, *p* = 0.04; ‘Distance’ (m), r = 0.7, *p* = 0.03; Figure 5B).

To further test the possible association of rats’ behavior with specific bacterial taxa, we used the ‘percentage of time spent in open space’ and the ‘percentage of time spent in the central zone’ from the EPM and OF tests, respectively. Using LEfSe, the microbiota composition of “low-anxiety rats” (eight samples taken from cages scoring the mean highest scores) was compared with “high anxiety rats” (eight samples taken from cages scoring the mean lowest scores). Additionally, comparisons for pre- and post-treatment samples were performed. Only bacterial taxa presenting similar and statistically significant associations with behavior in both analyses were further analyzed. The discriminating taxa identified by LEfSe were then directly correlated to the behavioral test scores (cage means). With EPM test scores, the abundance of *Adlercreutzia* (previously linked to depression in humans [55], and to anxiety and consumption of antidepressant medication in rodents [56,57,58]) and of *Turicibacter* (previously linked to depressive-like behavior in rats [59]) were significantly associated to increased anxiety-like behavior levels (Spearman’s ρ ‘% of Open Time’: *Adlercreutzia*, *p* = 0.002, r = -0.62; *Turicibacter*, *p* = 0.004, r = −0.6). In contrast, *Mogibacterium* and *Eubacterium* abundance was associated with decreased levels of anxiety-like behavior (Spearman’s ρ ‘% of Open Time’: *Mogibacterium*, *p* = 0.01, r = 0.53; *Eubacterium*, *p* = 0.03, r = 0.46). After FDR correction, the *p*-values were 0.008, 0.008, 0.013, and 0.03 for *Adlercreutzia*, *Turicibacter*, *Mogibacterium*, and *Eubacterium*, respectively. No statistically significant associations could be found when using the OF test scores, potentially because, in these tests, the effect size was smaller than in the EPM.

*Mogibacterium*’s relative abundance represented the best biomarker for oxytocin effectiveness, as it was significantly and positively associated with both “low-anxiety” behavior and oxytocin administration. Rats treated with oxytocin that then went on to display more “low-anxiety” behaviors in the EPM test also had relatively higher levels of *Mogibacterium relative* abundance (Spearman’s ρ for EPM Test parameters: ‘% of Open Time,’ r = 0.92, *p* = 0.001; ‘% of Open Entries,’ r = 0.92, *p* = 0.0008; ‘Number of Total Entries,’ r = 0.84, *p* = 0.006; ‘Closed Arm-Entries,’ r = 0.7, *p* = 0.06; Figure 5C).

## 4. Discussion

Precision, patient-adjusted therapy is critically needed in psychiatric medicine since drug responses can differ across individuals because of different pharmacokinetic and pharmacodynamics parameters across patients [60]. However, our current understanding of psychiatric diseases and drug mechanisms is generally too limited to achieve patient-specific therapy. In order for treatments to become more individualized, objective physiological parameters that assess the effectiveness of medication are needed. Microbiome studies offer an opportunity to find such parameters for some psychiatric drugs, as objective changes in the microbiota may reflect treatment efficacy [61]. The problem, however, is that microbiota are very sensitive to perturbations and are influenced by a wide variety of factors. For example, patients taking several classes of medications or with mood swings affecting their activity levels (such as depressed vs. manic in bipolar disorder) may experience changes in their microbiota as a result. These confounding factors compromise the use of the fecal microbiota features as biomarkers in psychiatry.

Nonetheless, this study aimed to be a proof of concept for the use of microbiota changes as indicators for drug intake. Using a multidrug environment with stress application in a rat model, we modeled some aspects of the psychiatric field environment. We observed that the microbiota composition shift and the relative abundance of the genus *Mogibacterium* were drug-specific changes in the microbiota. These changes were specifically induced by oxytocin and consistently associated with rat behavior, regardless of other drugs given or whether stress was applied.

Even though oxytocin is known to have a role in social bonding, sexual reproduction, and childbirth [62], today, it is medically used mostly for the augmentation of vaginal delivery [63]. However, as it is a profound anxiolytic and anti-stress factor in the brain [47], the use of oxytocin as an anxiolytic in preclinical and clinical research, though highly debated, points to the oxytocinergic system as a target for novel anxiolytics [64]. Oxytocin’s effect on human behavior was portrayed as relatively weak and inconsistent, suggesting its influence on behavior might be situation- or individual-dependent [65], but it was presumed that imbalance of the endogenous brain oxytocinergic system underlies the etiology of anxiety disorders, particularly those with a social component [47]. There is also conflicting evidence of oxytocin’s influence on the gut, especially gut motility. Oxytocin-treated rats, for example, previously showed either an increase [66] or a decrease [67,68] in bowel movement and emptying. This inconsistency could be partly due to the diverse effects of different responses in different parts of the GI tract, as oxytocin’s receptors are expressed across the entire GI tract in humans [69] and in rats [66,67,70]. In regard to the microbiota, although mouse studies have shown that the gut microbiota can influence oxytocin in the brain [71,72], there is little data on how exogenous oxytocin affects the microbiota.

Nevertheless, under oxytocin treatment, we observed a strong shift in the microbiota composition manifesting as a large UniFrac distance between pre- and post-treatment states that we referred to as a ‘microbiota composition shift.’ The magnitude of that shift was also positively associated with the rat’s anxiolytic-like scores in behavioral tests. Thus, we were able to define a parameter that may reflect the administration and effectiveness of oxytocin treatment in rats. Furthermore, we were able to pinpoint a specific bacterial genus, *Mogibacterium*, which showed an increase in relative abundance following oxytocin treatment and was associated with low-anxiety behavior in rats. This demonstrated that specific members of the microbiota can be associated with drug efficacy and not just drug intake and raises the hope that such biomarkers could be identified in the future in the human microbiome.

Our study also had several limitations. First, the study was performed on adult male rats, and while behavioral tests were conducted per individual animal, the microbiota data were collected per cage for Experiment 1. Therefore, to compare these two variables, the mean behavioral test score per cage was used, reducing sensitivity. Second, it should be acknowledged that, unlike oxytocin, citalopram did not produce a substantial shift in the microbiota, indicating that for some drugs, the correspondence between changes to the microbiota and efficacy may be low. Third, 16 S rRNA gene amplicon sequencing reveals taxonomy only, and one is limited in the ability to draw conclusions on functional changes in the microbiota, for example, the ability to produce bioactive metabolites that may affect host behavior or physiology. Future work should include shotgun metagenomics and metabolomics in order to have not just better chances of identification of microbiota-based indicators of treatment success but also to gain clues into the mechanism. Additionally, the effects on immunity should also be explored, as often microbiota shifts can change the levels of inflammation, which in turn can affect behavior. Fourth, rodent models are a useful starting point, but because animals are nearly identical in their genotype and environmental exposures, such studies cannot capture the large inter-individual variation in humans, which makes the identification of broadly applicable clinical biomarkers a challenging task.

## Data Availability

Data are available on Figshare - https://figshare.com/account/login#/projects/122474 (accessed on 5 September 2021).

## Supplementary Materials

The following are available online at https://www.mdpi.com/article/10.3390/microorganisms9091938/s1, Figure S1: Effects of different treatments on the rat fecal microbiota, Figure S2: Microbiota composition shift following various treatments, Figure S3: Effects of corticosterone and citalopram on the rat fecal microbiota, Figure S4: Bacterial genera significantly differentiating oxytocin-treated and non-treated groups
