# Supplementary material for: Specific Changes in the Mammalian Gut Microbiome as a Biomarker for Oxytocin-Induced Behavioral Changes
Source: Microorganisms. 2021 Sep 12;9(9):1938. doi: 10.3390/microorganisms9091938 (PMC8465812; doi:10.3390/microorganisms9091938)
Supplement: Supplementary file 1 [file microorganisms-09-01938-s001.zip › Revised Supplementary Figure S1.pdf]

Supplementary Figure S1.

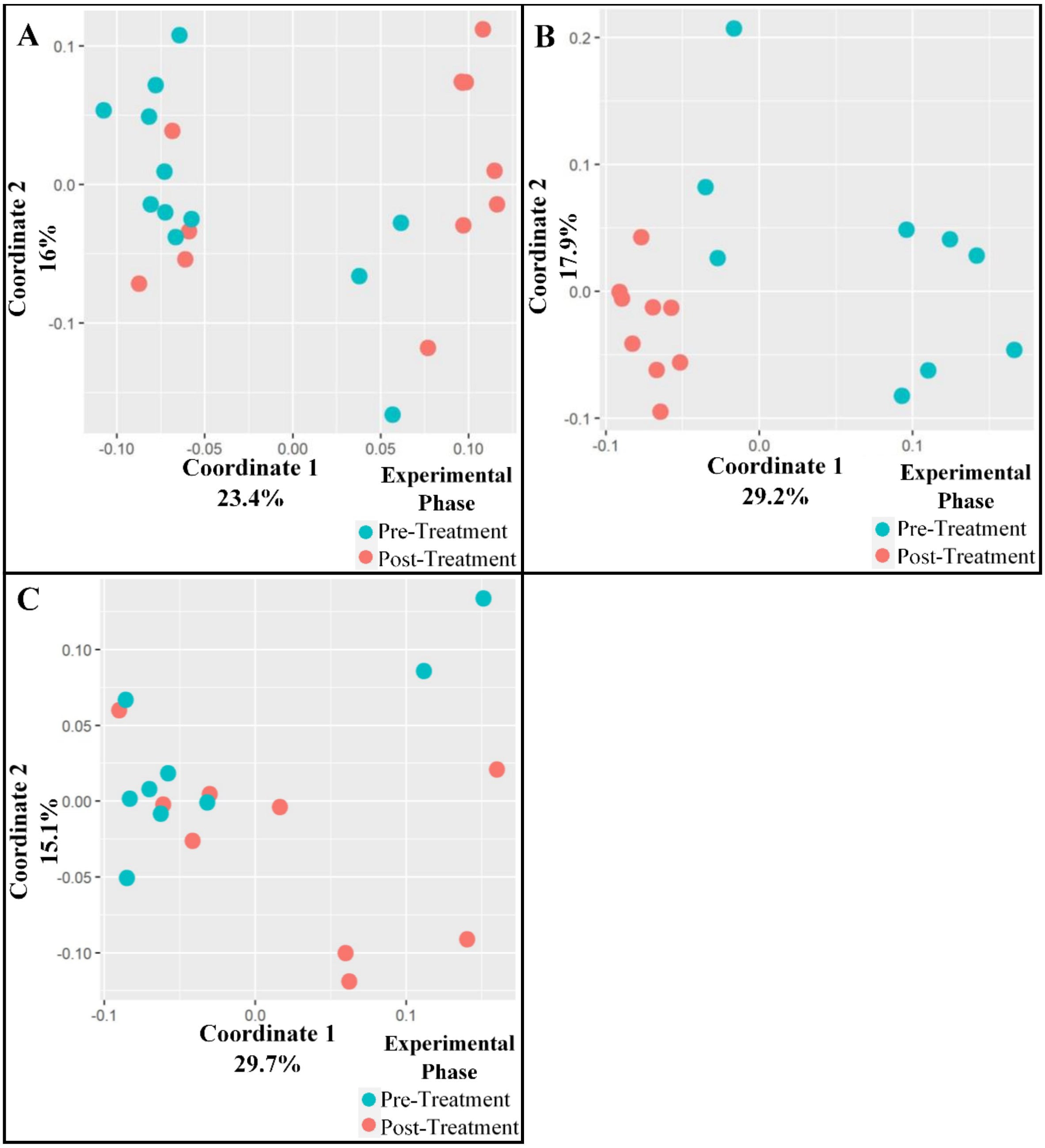

Supplementary Figure S1. **Pre and post treatment effect**. n; each point represents the microbiota composition of a single cage. **A:** Samples from the corticosterone treated-group only; pre-treatment (green) vs post-treatment (red) ANOSIM, **P=0.003, R=0.23**. **B:** Samples from the oxytocin treated-group only; pre-treatment (green) vs post-treatment (red) ANOSIM, **P=0.001, R=0.54**. **C:** Samples from the citalopram treated-group only; pre-treatment (green) vs post-treatment (red) ANOSIM, P=0.1, R=0.07.
