# Supplementary material for: Specific Changes in the Mammalian Gut Microbiome as a Biomarker for Oxytocin-Induced Behavioral Changes
Source: Microorganisms. 2021 Sep 12;9(9):1938. doi: 10.3390/microorganisms9091938 (PMC8465812; doi:10.3390/microorganisms9091938)
Supplement: Supplementary file 1 [file microorganisms-09-01938-s001.zip › Revised Supplementary Figure S2.pdf]

Supplementary Figure S2.

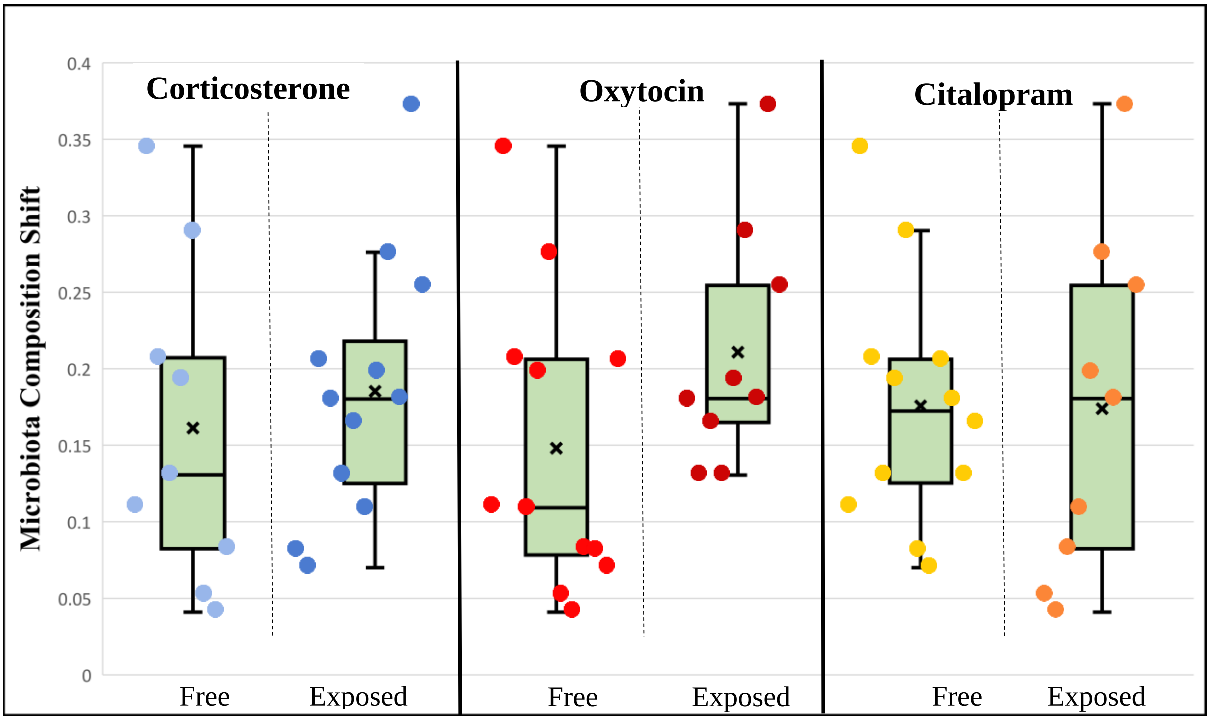

Su pplementary Figure S2. **Treatment effect on microbiota.** The Microbiota Composition Shift (weighted UniFrac distances between phase I (pre-treatment) and phase II (post-treatment) microbiota samples) n; each point represents a single cage.
