# Supplementary material for: Specific Changes in the Mammalian Gut Microbiome as a Biomarker for Oxytocin-Induced Behavioral Changes
Source: Microorganisms. 2021 Sep 12;9(9):1938. doi: 10.3390/microorganisms9091938 (PMC8465812; doi:10.3390/microorganisms9091938)
Supplement: Supplementary file 1 [file microorganisms-09-01938-s001.zip › Revised Supplementary Figure S3.pdf]

Supplementary Figure S3.

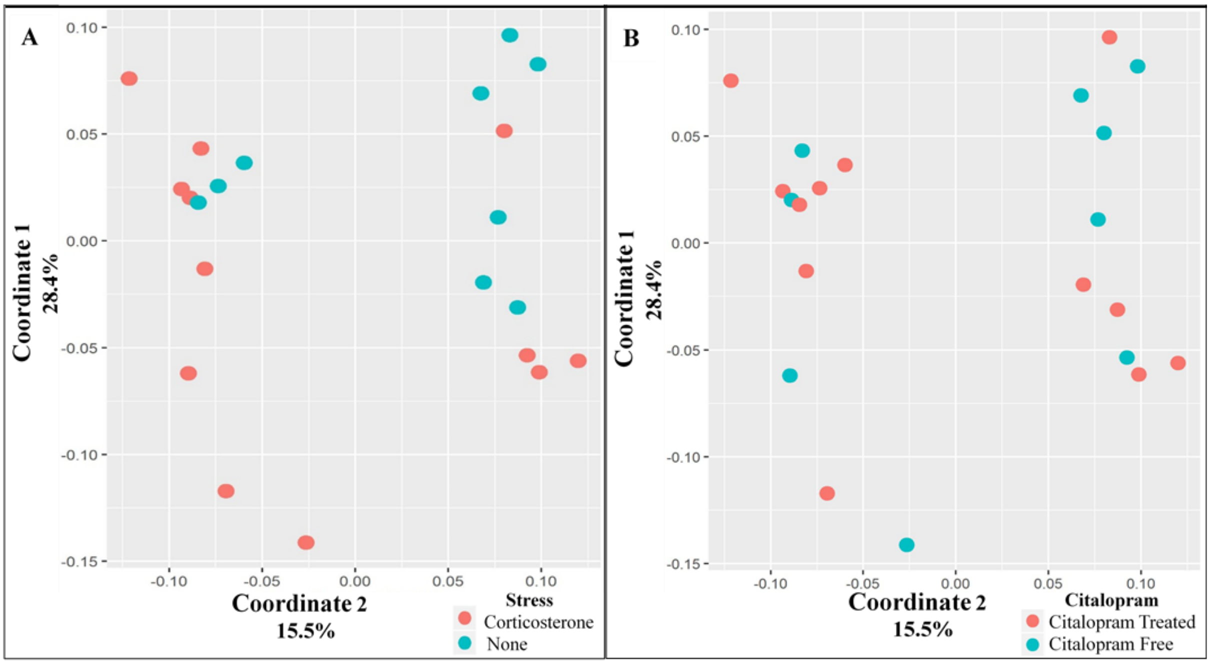

Supplementary Figure S3. **Treatment effect on microbiota.** PCoA of the unweighted UniFrac matrix of all – post-trea tment samples. **A.** Corticosterone, ANOSIM, **P=0.001**, **R=0.43**. **B.** Citalopram had no statistically significant effect.
