# Supplementary material for: Specific Changes in the Mammalian Gut Microbiome as a Biomarker for Oxytocin-Induced Behavioral Changes
Source: Microorganisms. 2021 Sep 12;9(9):1938. doi: 10.3390/microorganisms9091938 (PMC8465812; doi:10.3390/microorganisms9091938)
Supplement: Supplementary file 1 [file microorganisms-09-01938-s001.zip › Revised Supplementary Figure S4.pdf]

Supplementary Figure S4.

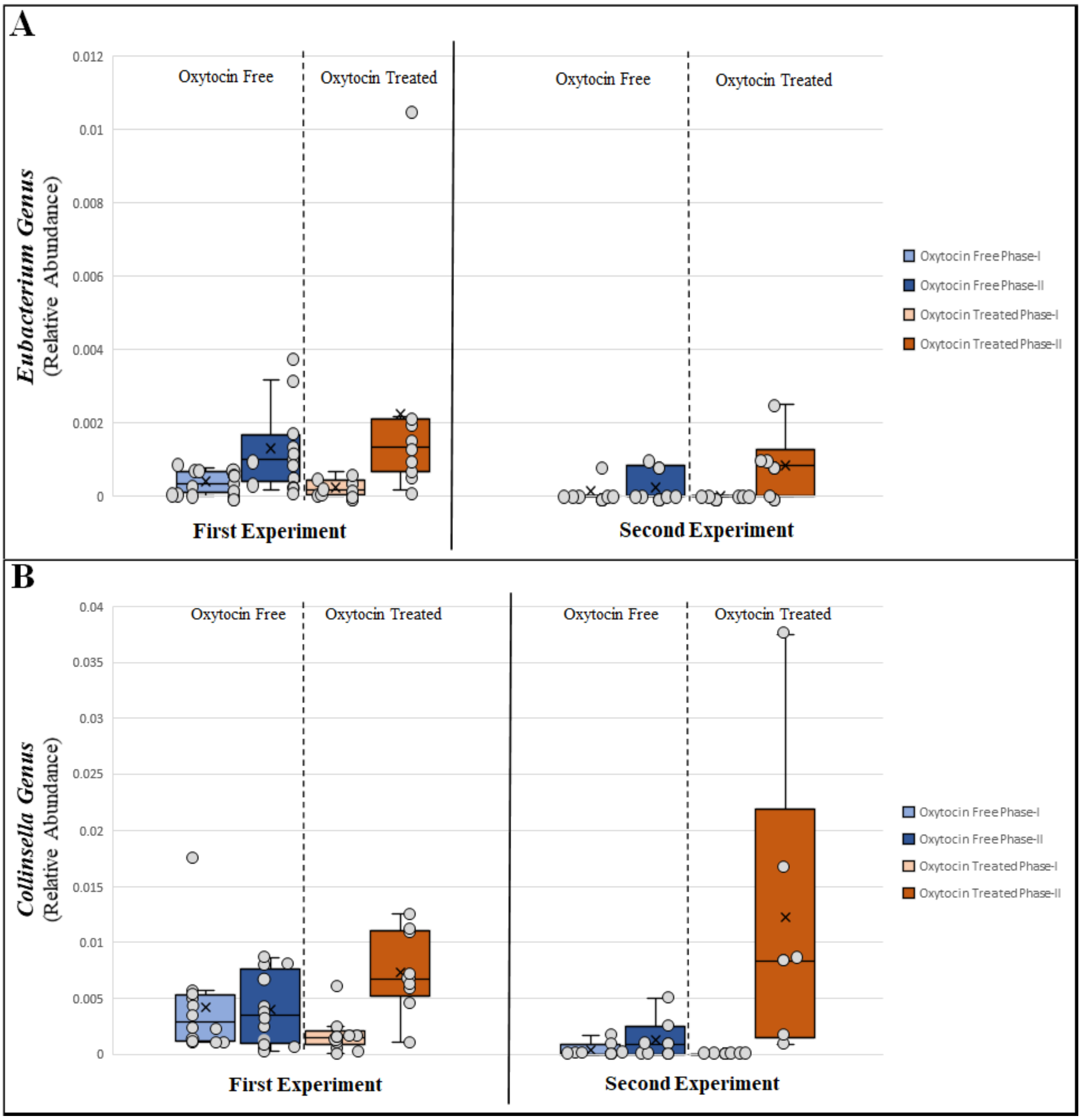

Supplementary Figure S4. **Bacterial genera significantly differentiating oxytocin-treated and non-treated groups.** First experiment: each circle represents a single cage pool. Second experiment: each circle represents a single animal. Phase-I: pre-treatment, phase-II: post-treatment. **A.** *Eubacterium*; **B.** *Collinsella*.
