# Supplementary material for: Specific Changes in the Mammalian Gut Microbiome as a Biomarker for Oxytocin-Induced Behavioral Changes
Source: Microorganisms. 2021 Sep 12;9(9):1938. doi: 10.3390/microorganisms9091938 (PMC8465812; doi:10.3390/microorganisms9091938)
Supplement: Supplementary file 1 [file microorganisms-09-01938-s001.zip › Supplementary Figure caption.pdf]

Supplementary Figure S1.

Effects of different treatments on the rat fecal microbiota. n; each point represents the microbiota composition of a single cage. **A:** Samples from the corticosterone treated-group only; pre-treatment (green) vs post-treatment (red) ANOSIM, **P=0.003, R=0.23**. **B:** Samples from the oxytocin treated-group only; pre-treatment (green) vs post-treatment (red) ANOSIM, **P=0.001, R=0.54**. **C:** Samples from the citalopram treated-group only; pre-treatment (green) vs post-treatment (red) ANOSIM, P=0.1, R=0.07.

Supplementary Figure S2.

Microbiota composition shift following various treatments. A. The Microbiota composition shift (weighted UniFrac distances between phase I (pre-treatment) and phase II (post-treatment) microbiota samples) n; each point represents a single cage.

Supplementary Figure S3.

Effects of cortiscosterone and citalopram on the rat fecal microbiota . PCoA of the unweighted UniFrac matrix of all – post-treatment samples. **A.** Corticosterone, ANOSIM, **P=0.001**, **R=0.43. B.** Citalopram had no statistically significant effect.

Supplementary Figure S4.

Bacterial genera significantly differentiating oxytocin-treated and non-treated groups. First experiment: each circle represents a single cage pool. Second experiment: each circle represents a single animal. phase-I: pre-treatment, phase-II: post-treatment.

A. *Eubacterium*; **B.** *Collinsella*.
